# Coadministration of fluoride, trimetaphosphate, and quercetin reduces dentin erosive wear *in vitro*

**DOI:** 10.1007/s00784-026-07177-z

**Published:** 2026-10-03

**Authors:** Isabela Maria Passarela Gomes, Alberto Carlos Botazzo Delbem, Tamires Passadori Martins, Isabela Ferreira da Silva, Lucas Fernando Oliveira Tomáz Ferraresso, Letícia Cabrera Capalbo, Marília Afonso Rabelo Buzalaf, Douglas Roberto Monteiro, Juliano Pelim Pessan

**Affiliations:** 1 School Dentistry, Department of Pediatric Dentistry and Public Health, São Paulo State University (UNESP), José Bonifácio, Araçatuba, SP 1193 – 16015-050 Brazil; 2 Division of Clinical Essentials and Simulation, University of Detroit Mercy School of Dentistry, Detroit, MI USA; 3 Bauru School of Dentistry, Department of Biological Sciences, University of São Paulo (USP), Bauru, SP Brazil; 4 Present Address: School of Dentistry, Department of Diagnosis and Surgery, São Paulo State University (UNESP), Rua José Bonifácio, 1193, Araçatuba, SP 16015-050 Brazil

**Keywords:** Tooth erosion, Fluoride, Polyphosphates, Flavonoids

## Abstract

**Objectives:**

To evaluate the effect of solutions containing quercetin (QC), sodium trimetaphosphate (TMP), and fluoride (F), in different combinations, on dentin erosive wear (DEW) in vitro.

**Materials and methods:**

Bovine root dentin blocks were randomly divided into seven experimental groups (*n* = 28/group): deionized water (control); 500 ppm F (500F); 1100 ppm F (1100F); 5000 ppm F (5000F); 1100 F + 0.03% QC (F + QC); 1100 F + 1% TMP (F + TMP); and 1100 F + 1% TMP + 0.03% QC (“EXP” - experimental). Blocks were treated twice/day and subjected to four daily erosive challenges for five days (0.05 M citric acid, pH 3.2, 90 s), either erosion only (ERO) or erosion plus abrasion by brushing (ERO + ABR). The samples were analyzed by profilometry and computed X-ray microtomography, and the data were analyzed by 2-way ANOVA followed by Fisher’s LSD *post hoc* test.

**Results:**

Under both conditions, a dose-response relationship was observed between F concentration and all variables, with the lowest values for 5000F and EXP. Under ERO, only 5000F showed lower DEW than 1100F; under ERO + ABR, all TMP and/or QC-containing solutions reduced DEW compared with 1100F, with a similar trend for mineral loss and depth of mineral loss, most notably for 5000F and EXP.

**Conclusions:**

Adding QC or TMP to 1100F produced modest additional protection, while their combination yielded a significant additive effect comparable to that of a solution with a five-fold higher F concentration.

**Clinical relevance:**

Possibility of reducing fluoride concentration in preventive formulations with an effect similar to that of high-concentration formulations against DEW.

## Introduction

The modern lifestyle, characterized by high consumption of acidic foods and beverages, has increased the prevalence of dental erosion, a non-bacterial chemical process that causes irreversible dissolution of dental hard tissues and reduces their resistance to mechanical challenges, resulting in erosive tooth wear (ETW) [1, 2]. Early diagnosis remains challenging, and lesions are often identified only at more advanced stages [1]. As erosion progresses to dentin, collagen fibers become exposed and susceptible to enzymatic degradation, making the use of antiproteolytic agents essential for the control of dentin erosive wear (DEW) [3].

Preventive strategies for managing DEW include dietary counseling, stimulation of salivary flow, dietary modification, appropriate oral hygiene practices, and fluoride (F) therapy [4]. Unlike dental caries, however, the treatment of erosive tooth wear requires higher F concentrations and more frequent applications [5, 6] since erosive challenges cause immediate and irreversible superficial mineral loss due to a sharp decrease in pH. Fluoride therapy provides additional minerals (CaF₂) to enamel surfaces, reducing the degree of dissolution after acidic challenges; however, its inappropriate use may increase the risk of toxicity [6].

To enhance the beneficial effects of F in both home-use and professional products, alternative strategies have been explored, including the addition of phosphate salts, which have shown important synergistic effects with F. The incorporation of sodium trimetaphosphate (TMP) into F-containing products has been shown to significantly increase their protective and therapeutic effects against ETW in both enamel and dentin [7–9], involving mechanisms related to TMP’s ability to reduce acid diffusion, in addition to changes in surface free energy and the nucleation of ionic species that take part in tooth protection and rehardening [10–12].

In addition to phosphates, quercetin (QC) is a flavonoid found in various fruits and vegetables that exhibits anti-inflammatory, antioxidant, and anti-aggregatory properties and has been increasingly investigated in the past few years [13]. In the context of DEW, QC reduces collagen degradation by inhibiting matrix metalloproteinases (MMPs), thereby decreasing demineralization and enhancing dentin protection [14–17]. An in vitro study demonstrated that solutions containing QC combined with sodium hexametaphosphate (HMP), with or without F, promoted greater MMP inhibition and a protective effect on eroded dentin when compared with the positive control (1100 ppm F) [3].

This study is novel in investigating the combination of F, TMP, and QC to evaluate possible additive effects that are still poorly explored in dentin. Furthermore, it includes the simulation of toothbrushing abrasion, making it closer to real clinical conditions and differentiating it from previous studies that did not consider this factor. In view of the above, the objective of this study was to evaluate the effect of solutions containing F, TMP, and QC in different combinations, associated or not with abrasion by toothbrushing, in vitro. The primary null hypothesis was that the addition of TMP and/or QC to a fluoridated solution would not influence the effect of F on DEW. The secondary null hypothesis was that the treatment solutions would not affect the integrated mineral loss or the depth of mineral loss of the remaining dentin.

## Methodology

### Experimental design

**Fig. 1:**
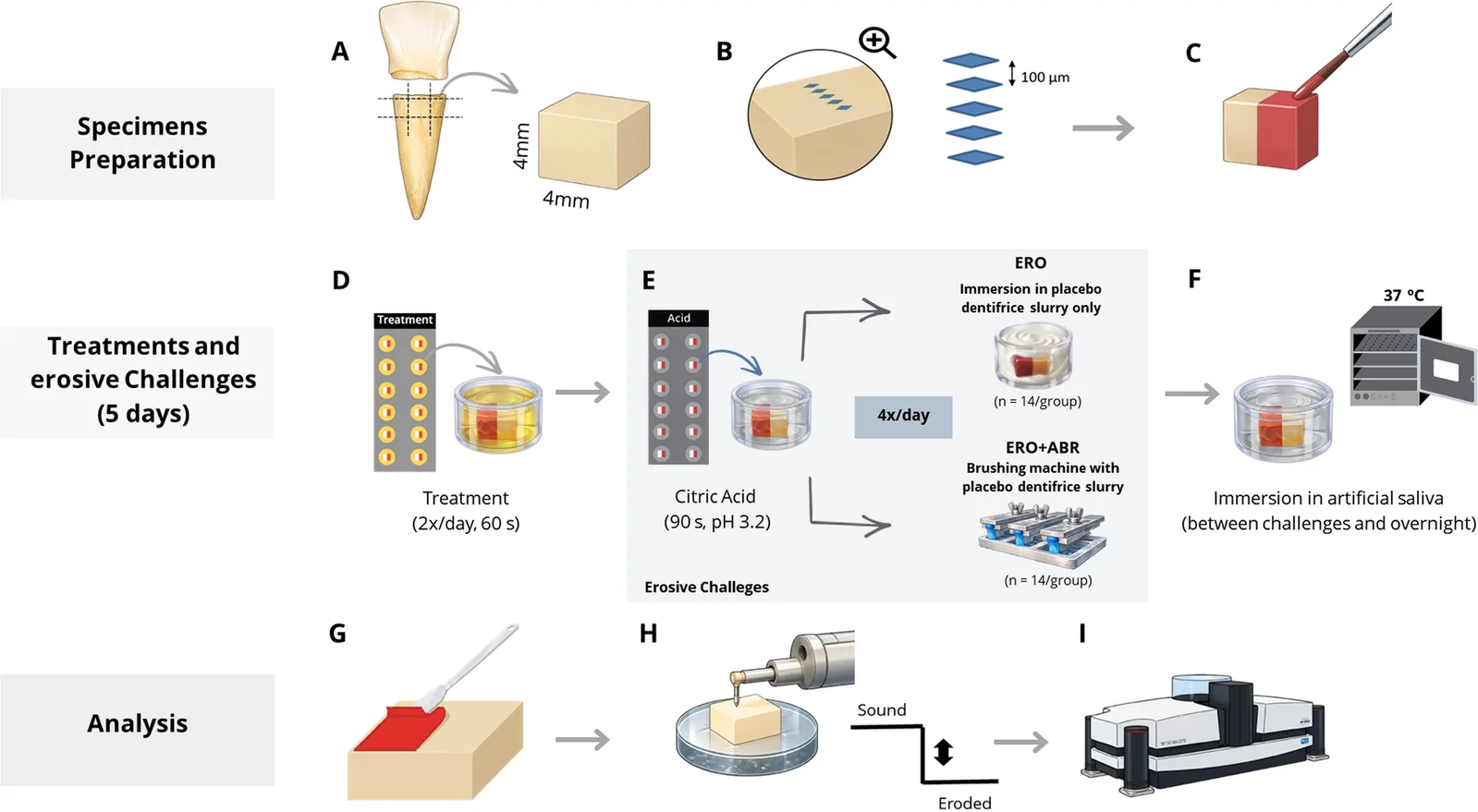
Flowchart summarizing the study design. **A** Dentin blocks (4 × 4 mm) obtained from bovine roots; (**B**) Initial surface microhardness analysis for randomization; (**C**) Half of the block’s surface coated with acid-resistant varnish, creating control and test areas; (**D**) Specimens treated twice daily for 1 min with the solutions; (**E**) Four daily ERO and ERO + ABR challenges for 5 days; (**F**) Storage in artificial saliva between challenges and overnight; (**G**) Varnish removal; (**H**) profilometric analysis; (**I**) computed X-ray microtomography analysis

This study was submitted to and approved by the Animal Use Ethics Committee (CEUA), São Paulo State University (UNESP), School of Dentistry, Araçatuba, for the collection and use of bovine teeth (Protocol nº 570–2024). Bovine dentin blocks (*n* = 196) were sequentially polished and selected based on surface microhardness (SH) (overall mean = 80.11 KHN; standard deviation = 7.81; 95% confidence interval = 0.87) [18]. Specimens were stratified and assigned to seven experimental groups (*n* = 28 per group), with selection criteria restricted to values between 79.24 KHN (minimum) and 80.99 KHN (maximum) to balance initial hardness means and standard deviations across all groups. Each group was subsequently divided into two sub-groups according to the challenge condition (*n* = 14/group): erosion (ERO) and erosion plus abrasion (ERO + ABR). The treatment solutions were: (1) deionized water (negative control; pH 5.62); (2) 500 ppm F (500F; pH 6.44); (3) 1100 ppm F (1100F; pH 6.94); (4) 5000 ppm F (5000F; pH 7.49); (5) 1100F + 0.03% QC (F + QC; pH 6.73); (6) 1100F + 1% TMP (F + TMP; pH 5.71); and (7) 1100F + 1% TMP + 0.03% QC (F + TMP+QC – “EXP”; pH 5.71). Half of the surface of each block was covered with an acid-resistant varnish (Risqué Savoy Indústria de Cosméticos S.A., São Paulo, Brazil) to serve as a reference area. The specimens were treated with one of the solutions twice daily for 1 min and subjected to four daily erosive challenges for five days. Half of the blocks in each group were immersed in a placebo toothpaste for 15 s (ERO), while the remaining specimens were subjected to brushing abrasion (ERO + ABR). Profilometry and computed X-ray microtomography (micro-CT) were performed sequentially on the same specimens. Between measurements, specimens were stored in Eppendorf tubes containing a deionized water-moistened gauze.” (Fig. 1) [19].

### Dentin blocks preparation and sample size calculation

Dentin blocks (4 × 4 × 2 mm) were obtained from the roots of one hundred ninety-six bovine central incisors (one block per tooth). Polishing was performed with 320, 600, 800, and 1200 grinder polishers (CARBIMET PaperDiscs, 30-5108-320, BUEHLER), with a final polish using a felt disc (Buehler Polishing Cloth 40–7618) moistened with a 1-µm diamond polishing suspension (Extec Corp., Enfield, CT, USA) [20]. The blocks were then selected based on surface microhardness (60.0–85.0 KHN; Shimadzu HMV-2000, Shimadzu Corporation, Kyoto, Japan), using a Knoop diamond indenter (10 g, 10 s) [18]. The sample size was based on a pilot study with *n* = 5/group, according to which 13 blocks would be required to detect significant differences in mean dentin loss of blocks treated with 1100F (positive control) and EXP under the condition ERO + ABR, considering mean difference = 4.7, standard deviation = 3.11, power of 80% (α = 0.05) and total number of groups of 7 (SigmaPlot software, Systat Software Inc., San Jose, USA, version 12.0). Due to the possibility of losses during the processing of the specimens, 14 blocks were included in each group × challenge.

### Preparation of treatment solutions

The concentrations of F, TMP, and QC in the solutions were determined based on a previous in vitro study that evaluated the effect of the combination of 1100 ppm F + 0.03% QC + 1.0% HMP on DEW [3]. The experimental solutions were prepared using F, TMP, and QC (Sigma-Aldrich Co., St. Louis, USA; CAS Nº 7681-49-4, 7785-84-4, and 117-39-5, respectively). To prepare the solutions containing QC, it was first dissolved in pure ethanol, followed by vortexing agitation and heating in a water bath at 37 °C for 15 min and subsequently added to deionized water under stirring [17, 21]. Ethanol acted exclusively as a vehicle to incorporate the hydrophobic flavonoid into solution [3] and was excluded from the control and remaining experimental groups, as pure ethanol alone has been shown to have no protective or detrimental influence on dentin demineralization or collagen preservation compared to water [15]. The solutions were then analyzed using a specific fluoride ion electrode, previously calibrated with five standards (2.0, 4.0, 8.0, 16.0, and 32 µg F/mL) [22]. The data obtained in mV were converted to µg F/mL to verify the actual fluoride concentration in the solutions. The mean (standard deviations) fluoride concentrations of the solutions were 13 (2.4), 527 (12.32), 1117 (22.5), 5287 (106.2), 1076 (83.4), 1069 (5.3), 1079 (34.0) ppm F, respectively, for the control; 500F; 1100F; 5000F; F + TMP; F + QC and EXP.

### Treatment of blocks and erosive challenges

The blocks were treated with their respective solutions (4 mL) for 1 min, twice a day, before the first and third erosion challenges. The blocks were then washed with deionized water, dried with absorbent paper, and subjected to four daily erosion challenges at room temperature (25 °C), for a period of five days (ERO, immersion in 0.05 M citric acid (Synth^®^, Brazil), pH 3.2, 90 s/cycle, stirring 35/min (Shaker SK-300, São Paulo, Brazil)) [19]. Half of the blocks (*n* = 14/group) were immersed in placebo toothpaste slurry for 15 s, so that they were only subjected to erosive challenges (ERO). The other half of the samples were subjected to abrasion (ERO + ABR) using a brushing machine (axial load of 250 g, 5 back-and-forth movements/s, 15 s; Elquip Maq Escovação, São Carlos, Brazil) with placebo toothpaste slurry [19, 23]. Between each erosion challenge, the blocks remained at least for 2 h in artificial saliva and overnight (1.5 mmol.l^− 1^ Ca(NO3)2.4H2O; 0.9 mmol.l^− 1^ NaH2PO4.2H2O; 150 mmol. l^− 1^ KCl; 0.1 mol.l^− 1^ Tris buffer; pH 7.0; 37 °C, no agitation) [3].

### Analysis of dentin loss

The acid-resistant varnish layer was carefully removed from the control surface with a spatula and a cotton swab soaked in acetone. Dentin loss was determined in relation to the healthy surface using profilometry analysis (Mahr Surf GD25 - Mahr, Göttingen, Alemanha), in which the tip of the roughness tester moved from the sound surface of the specimens toward the eroded area. Five readings were taken at 0.5 mm intervals in the center of the sample to calculate the average wear for each block [6, 24].

### Analysis of the mineral content of dentin by computed X-ray microtomography

Dentin blocks were analyzed by computed X-ray microtomography (Skyscan 1272, Bruker, Kontich, Belgium) operated at 90 ĸV, 110 µA, 0.5 mm aluminum filter, 4 μm spatial resolution, 0.600º rotation step, and random movement at 10. The image projections were reconstructed using the NRecon software (version 1.6.10.2, Skyscan1272; Bruker-MicroCT) with smoothing at 5, ring artifact correction at 12, and hardening correction at 52% [25].

After reconstruction, two-dimensional (2D) virtual slices in the sagittal and coronal planes were acquired using Data Viewer software (Skyscan1272). The group of 2D slices was imported into ImageJ software (version 1.47v) to perform a Z-projection of the standardized volume of interest (VOI). The average intensity projection algorithm was applied to collapse the sequential 2D slices into a single representative bi-dimensional image. This process allowed a side-by-side structural comparison and visual contrast between the treated and untreated dentin areas. Subsequently, a specific region of interest (ROI) was defined for each area, using the outer surface of the untreated dentin as the external boundary and extending to a depth limit of 700 μm. This delimited volume was used to produce a general profile of mineral concentration (gHAp × cm − 3) as a function of depth (µm), based on a calibration curve (linear regression, R2 = 0.9985) calculated with reference standards (0, 0.25, and 0.75 gHAp × cm–3). The integrated area under the mineral concentration profile was calculated for both treated and untreated areas using the trapezoidal rule in GraphPad Prism software (version 8.00). The integrated mineral loss (ΔIML; gHAp × cm⁻³ × µm) was then determined by subtracting the integrated value of the treated area exposed to erosive cycles from that of the untreated area [25]. Additionally, the depth of mineral loss (µm) was determined by measuring down to a fixed depth of 700 μm, using the external surface of the untreated dentin as the baseline reference.

### Statistical analysis

SigmaPlot software (Systat Software Inc., San Jose, USA), version 12.0, was used, and the significance level was set at 5%. Although the data did not meet the assumptions of normality and homoscedasticity, parametric analysis was maintained based on the proven robustness of ANOVA against distributional violations in dental data, which preserves actual Type I error rates and statistical power in factorial designs [26]. Thus, data were submitted to 2-way ANOVA followed by Fisher’s LSD *post hoc* test.

## Results

### Determination of DEW

**Fig. 2:**
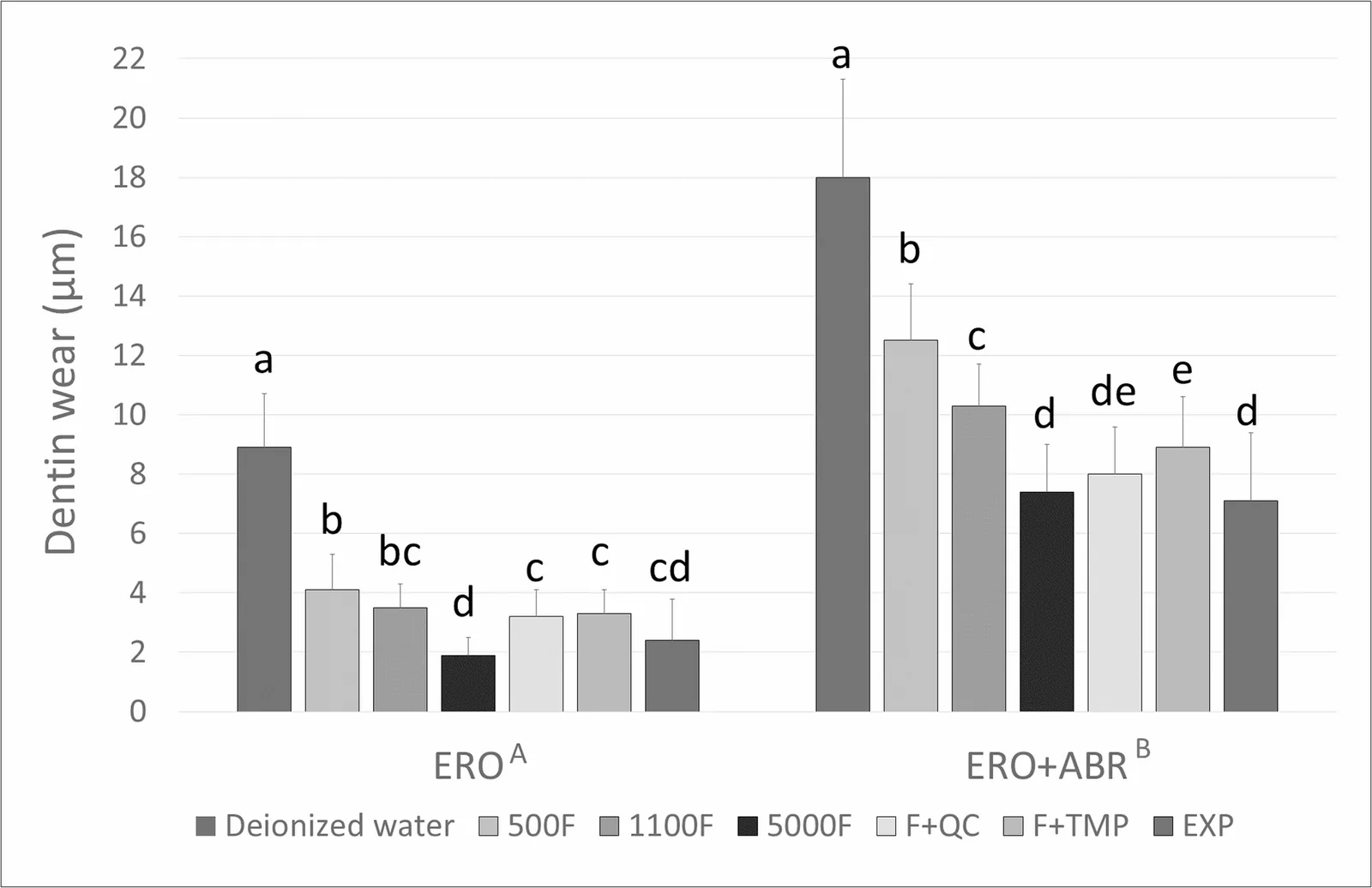
Dentin wear (µm) after treatment with the solutions and exposure to erosive or erosive+abrasive challenges. Lowercase letters indicate significant differences between groups within each challenge; uppercase letters indicate significant differences between challenges within each group (2-way ANOVA followed by Fisher’s LSD *post hoc* test, *p* < 0.05, *n* = 14 per treatment × challenge condition. Deionized water (negative control); 500 ppm F (500F); 1100 ppm F (1100F); 5000 ppm F (5000F); 0.03% QC + 1100 F (F + QC); 1100 F + 1% TMP (F + TMP); 1100 ppm F + 1% TMP + 0.03% QC (F + TMP+QC – “EXP” - experimental)

Figure 2 shows the mean (SD) dentin loss (µm) according to the experimental solutions. Statistical analysis revealed significant differences among the treatment groups (F = 96.2, *p* < 0.001), between the challenges (F = 730.7, *p* < 0.0001), as well as for the interaction between these variables (F = 7.8, *p* < 0.001), with power = 1.0 for groups, challenges, and the interaction between both variables. Under both conditions, a dose-response relationship was observed between F concentration in solutions without other active agents (deionized water, 500F, 1100F, and 5000F) and protection against DEW. The lowest wear values were observed for 5000F and EXP. Under the ERO condition, only 5000F showed a greater protective effect than 1100F, whereas EXP showed a similar effect to both 1100F and 5000F. As for ERO + ABR, all experimental solutions containing TMP and/or QC provided greater protection than 1100F, with no significant differences among 5000F, F + QC, and EXP. All values obtained for ERO were significantly lower than those for ERO + ABR in all pairwise comparisons.

### Determination of mineral content of dentin and erosive depth of mineral loss

For both variables, significant differences were observed among the groups, between the challenges, and for the interaction between these variables (*p* < 0.001), with power = 1.0 for all the variables except for the interaction between groups and challenges concerning depth of mineral loss (power = 0.81). The data for both variables followed a similar trend to that described for DEW, including a dose-response relationship between F concentration in solutions without other active agents and both mineral content and depth of mineral loss, and a superior overall performance of 5000F and EXP compared with the other groups (Table 1).

**Table 1:** Mean values of integrated mineral loss and depth of erosive lesions under different challenge conditions

Groups	Analyzes			
	Integrated mineral loss (gHAp × cm^− 3^ × µm)		Depth of mineral loss (µm)	
	ERO	ERO + ABR	ERO	ERO + ABR
Deionized water	-2.37^A, a^	-4.42^B, a^	165.8^A, a^	214.5^B, a^
	(0.33)	(0.33)	(18.4)	(31.6)
500F	-1.99^A, b^	-3.75^B, b^	151.0^A, b^	194.2^B, b^
	(0.16)	(0.34)	(16.5)	(28.1)
1100F	-1.64^A, c^	-2.66^B, c^	137.1^A, bc^	160.8^B, c^
	(0.17)	(0.21)	(16.6)	(13.3)
5000F	-1.18^A, d^	-2.19^B, d^	109.2^A, d^	119.7^A, d^
	(0.13)	(0.10)	(16.3)	(22.8)
F + TMP	-1.48^A, ce^	-2.40^B, ef^	121.0^A, d^	145.1^B, ce^
	(0.10)	(0.27)	(17.7)	(29.8)
F + QC	-1.44^A, ef^	-2.45^B, e^	123.5^A, cd^	145.1^B, e^
	(0.13)	(0.30)	(16.8)	(29.8)
EXP	-1.29^A, df^	-2.27^B, df^	111.2^A, d^	126.9^A, d^
	(0.09)	(0.31)	(14.8)	(13.9)

Lowercase letters indicate significant differences among groups within each challenge; uppercase letters indicate significant differences between challenges within each group. Values in parentheses represent standard deviations from the means (two-way ANOVA and Fisher’s LSD test, *p* < 0.05, *n* = 14 per treatment × challenge condition). Deionized water (negative control); 500 ppm F (500F); 1100 ppm F (1100F); 5000 ppm F (5000F); 1100F + 0.03% QC (F + QC); 1100F + 1% TMP (F + TMP); 1100F + 1% TMP + 0.03% QC (F + TMP+QC – “EXP” - experimental)

## Discussion

This study evaluated the effects of F, TMP, and QC, in different combinations, on DEW under either ERO or ERO + ABR challenges in vitro. Overall, the results demonstrated that the protective effect against DEW was influenced by both F concentration and the presence of additional active agents, with more pronounced differences observed under the ERO + ABR condition. While a dose-response relationship was evident for solutions containing F alone, the addition of TMP and/or QC to 1100F resulted in additional protective effects, with significant differences from 1100F under the more aggressive challenge, with similar trends observed in both integrated mineral loss and depth of mineral loss. Thus, both null hypotheses were partially rejected.

It is widely known that although F can reduce erosive tooth wear compared with no treatment, the protective layer it forms (primarily CaF_2_-like deposits) may be relatively labile during intense or repeated acidic challenges, and significant protection often requires high concentrations, frequent application, or specialized formulations beyond typical home use [5, 6]. In the present study, the results demonstrated significant differences between the negative control group and the groups containing active agents under both ERO and ERO + ABR conditions, and a dose–response relationship was observed among the solutions containing F alone, indicating that greater F availability favors the formation and replenishment of CaF_2_-like deposits and enhances the action of calcium and phosphate present in the artificial saliva, promoting surface rehardening and increased resistance to acid challenges [19].

The similar performance of 500F and 1100F under ERO might be attributed to the lower intensity of the erosive challenge, which preserves part of the organic matrix and the softened mineralized tissue. In the absence of abrasion, this collagen layer acts as a physical barrier to acid diffusion, ensuring moderate protection even at low F concentrations [6, 11]. In contrast, under ERO + ABR conditions, the repeated association between acid softening and mechanical stimulation compromises the structural stability of the surface, even though the organic matrix is not fully removed, as described in previous studies [27]. In this scenario, the protective role of the matrix may become less relevant, and treatment efficacy begins to depend on the formation of more stable mineral deposits, such as fluorapatite-like compounds [11]. Under these conditions, the dose-response pattern became more pronounced, with significant differences between 500F and 1100F and superior performance of the higher concentration or combined formulations. Wear was approximately twice as high as under isolated ERO, confirming that abrasion potentiates tissue loss, in agreement with previous studies [9].

Under ERO + ABR conditions, the solutions containing F + TMP demonstrated significant protective effects against DEW, indicating that the association between fluoride and TMP becomes particularly relevant under more aggressive challenges. This finding is consistent with in vitro evidence showing that the positive effects of the association between F and TMP are attributed to the adsorption of TMP to collagen and hydroxyapatite, favoring the deposition of calcium phosphate apatite [7, 11, 28, 29]. These mechanisms are especially important when abrasion removes the softened surface layer, making mineral reservoirs and matrix stabilization critical for tissue preservation. This effect is further enhanced when TMP is used at the nanometric scale in both enamel and dentin [8, 9, 30]. Furthermore, the combination of both active agents was shown to reduce mineral loss and lesion depth compared with F alone, while also promoting inhibition of MMP-2 and MMP-9 [11].

QC is a polyphenolic flavonoid capable of modulating the dentin matrix and contributing to erosion prevention through the inhibition of MMPs and the reduction of collagen degradation. Its polyphenolic structure, particularly due to the presence of phenolic hydroxyl groups, may favor the formation of stable hydrogen bonds with the side chains of hydroxyl, carboxyl, amine, or amide groups of collagen molecules, potentially promoting cross-linking processes. These interactions are associated with the changes observed in the amide I and amide III bands in spectroscopic analyses, suggesting possible structural modifications of collagen after treatment [15, 31, 32]. Previous studies have shown that, when used alone, its action provides only modest protection. However, when associated with a phosphate salt (HMP) and F, QC at a concentration of 0.03% exhibits superior protective effects, demonstrating an additive action among these agents [3]. In the present study, treatment with F + QC and EXP promoted marked reductions in DEW, especially under the ERO + ABR condition, presenting a similar performance to the 5000F group and superior performance to the F + TMP group. These results can be mainly attributed to the effect of QC on dentin tissue, particularly due to its ability to preserve the organic matrix and modulate MMP activity [32]. When associated with F and TMP, QC contributed to an increased mineral content, representing a promising alternative for formulations targeting both dental tissues, with TMP exerting effects on both enamel and dentin [7, 11] and QC predominantly on dentin [15, 32].

The overall superior performance of the EXP group was statistically similar to that observed in the 5000F group and can be attributed to the combined action of F, TMP, and QC on the inorganic and organic phases of dentin. While F provides essential mineral protection and rehardening through surface CaF_2_ deposition [19], TMP, in turn, due to its high affinity for hydroxyapatite, enhances the action of F, since the F-TMP interaction promotes significant mineralization, with deposition of approximately 20–40 μm in the outer dentin [10, 29]. Furthermore, a study demonstrated that QC can penetrate approximately 25–30 μm into dentin, preventing erosion and abrasion by enhancing the mechanical properties of the organic matrix; its penetration may be greater in demineralized dentin due to increased porosity [32]. Taken together, enhanced mineral deposition and preservation of the organic matrix likely explain the additive protection observed for the EXP formulation, yielding performance comparable to that of a high-concentration fluoride solution. Nevertheless, despite these complementary mechanisms, EXP did not uniformly outperform all experimental groups across every evaluated parameter. This variation may stem from potential competitive chemical interactions among F, TMP, and QC when coadministered, as the binding kinetics and surface adsorption of these active agents onto the dentin matrix can vary depending on the specific outcome measured [3]. Moreover, since this represents the first investigation of this tripartite combination, fine-tuning the concentration ratios of each component in future dose-response studies will be essential to fully optimize their synergistic potential against erosive and abrasive challenges.

A previous study showed that QC significantly inhibits MMP-2 and MMP-9, reaching complete inhibition when associated with F and HMP [3]. Since this study was conducted in vitro using polished dentin blocks, the endogenous activity of metalloproteinases may be reduced compared to that under clinical conditions [6]. Quercetin is described in the literature as an MMP inhibitor, primarily acting through the chelation of zinc present in the active site of these enzymes, which reduces their degradative activity [32, 33]. Thus, although we did not directly measure this action, it is plausible that in an oral environment, where MMPs are more active, the effect of quercetin may be even more promising.

Due to the irreversible dissolution of the dentin surface in erosion, the formation of a more mineralized external layer is paramount for protecting the tissue against further acid challenges [6]. The analysis of the mineral content of the remaining eroded dentin by computed X-ray microtomography proved to be an essential complementary tool, broadening the understanding of treatment effects by characterizing the condition of dentin that persisted after repeated erosive and abrasive challenges [11]. In the control group, mineral modification between cycles depended primarily on ions from the artificial saliva, whereas the treated groups were continuously exposed to F, TMP, and/or QC, which could interact with the residual substrate. This repeated exposure likely promoted varying degrees of mineral preservation, partial rehardening, and stabilization of the organic matrix, depending on the active agent. Less mineral loss and a shallower depth of loss were observed for the 5000F and combined treatments, followed by 1100F, 500F, and deionized water, confirming a clear dose-response trend.

The erosive protocol used in the present study may be considered too aggressive, as it involved prolonged acid exposure within short time intervals [3, 19, 23]. In contrast, erosive tooth wear in the clinical scenario occurs gradually and continuously over time, leading to cumulative loss of mineralized structure. This harsh protocol, combined with the use of an in vitro model using bovine dentin, represents an important limitation, as the results cannot be directly extrapolated to clinical reality. Also, the protocol did not account for interactions of the treatments with human saliva, which may influence the formation of CaF₂-like deposits, mineral precipitation, tissue rehardening, and stabilization of the organic matrix. However, the protocol was intended to ensure maximum methodological control, providing proof-of-principle data on the actual effects of the active agents on DEW. Future studies incorporating human dentin, in situ or in vivo conditions, and realistic saliva exposure are required to confirm these findings and assess the true clinical applicability of the tested formulations.

## Conclusion

The combination of QC, TMP, and F demonstrated an additive protective effect against dentin erosive wear in vitro, with performance comparable to that of the 5000F solution, showing potential as an alternative to high-fluoride formulations in preventive strategies.

## Data Availability

Datasets related to this article are available upon request to the corresponding author.
